# Novel, Low Cost, Highly Effective, Handmade Steroid Pellets for Experimental Studies

**DOI:** 10.1371/journal.pone.0064049

**Published:** 2013-05-15

**Authors:** Ana Sahores, Guillermina M. Luque, Victoria Wargon, María May, Alfredo Molinolo, Damasia Becu-Villalobos, Claudia Lanari, Caroline A. Lamb

**Affiliations:** 1 Institute of Experimental Biology and Medicine (IBYME), CONICET, Buenos Aires, Argentina; 2 Oral and Pharyngeal Cancer Branch, National Institute of Dental and Craniofacial Research, National Institutes of Health, Bethesda, Maryland, United States of America; Baylor College of Medicine, United States of America

## Abstract

The basic component of Silastic® glue (Dow Corning) used to prepare Silastic® pellets is polydimethylsiloxane. This compound is also present in other commercial adhesives such as FASTIX® (Akapol SA) that are available in any store for that category. In the present study we developed low cost, easy to prepare handmade steroid pellets (HMSP) by mixing 17β-estradiol, progesterone or other synthetic steroids with FASTIX® adhesive. We assessed serum levels of 17β-estradiol, progesterone, prolactin and luteinizing hormone in ovariectomized mice treated for 24 and 48 h or 7, 14 and 28 days with 20 µg or 5 mg of 17β-estradiol or 5 mg progesterone HMSP. We found a time dependent and significant increase in the levels of both natural hormones, and a downregulation of serum luteinizing hormone levels, while both 17β-estradiol doses increased serum prolactin. Uterine weights at sacrifice and histological examination of the uteri and the mammary glands correlated with estrogen or progestin action. Finally, we evaluated the biological effects of HMSP compared to commercial pellets or daily injections in the stimulation or inhibition of hormone dependent mammary tumor growth, and found that HMSP were as effective as the other methods of hormone administration. These data show that HMSP represent a useful, low cost, easily accessible method for administering steroids to mice.

## Introduction

Steroid hormones play key roles in a broad range of human physiological and pathological events [1], [2]. Rodents, mainly rats and mice are normally used to elucidate the effects of steroids in reproductive tissues. To avoid the cyclic influence of ovarian hormones, rodents are usually ovariectomized and steroid hormones replaced. Currently, different methods of delivery have been developed such as: daily subcutaneous (sc) injections [3], [4], sc slow-release pellets such as the ones provided by Innovative Research of America [3], [5] and Hormone Pellet Press [6], sc Silastic pellets [7], [8] or capsules [4], [5], [9] and, more recently, peroral administration [5], [10]. The clearest advantage of the pellets is that they are easily administered and do not require daily attention, although the major drawback of most pellets is their high cost [10]. Polydimethylsiloxane (PDMS) is a silicon-based organic polymer present in silicone capsules and silicone adhesive (Silastic®, Dow Corning). It has proven useful for preparing steroid capsules or pellets, because Silastic® allows steroids to pass through its walls, providing a means of chronic administration of the drugs for long periods of time [8], [9] and its chemical inertness avoids inflammatory reactions [11]. This component is also present in other low cost, commercially available adhesives such as FASTIX® (Akapol SA, Argentina).

The aim of the current study is to present and validate a new, effective, low cost procedure of preparing pellets in which steroids are mixed with a PDMS-containing adhesive. We evaluated the effect of these handmade steroid pellets (HMSP) on serum hormone levels, uterine weight and mammary tumor growth in ovariectomized mice.

## Materials and Methods

### Animals

Female BALB/c mice (Animal facility, IByME) were fed *ad libitum* and kept in air-conditioned rooms at 20±2°C with a 12 h light-dark period.

### Ethics Statement

Animal care and manipulation was in agreement with institutional guidelines, which are in accordance with the Guide for the Care and Use of Laboratory Animals [12]. This study was approved by the IByME Ethics Committee.

### Pellet Preparation

For HMSP preparation: progesterone (Pg), 17-β-estradiol (E2), medroxyprogesterone acetate (MPA) or mifepristone (MFP) all from Sigma Co. (St Louis, MO, USA), were thoroughly mixed with the PDMS-containing synthetic adhesive (FASTIX®). For Pg 5 mg we used 300 mg Pg +1.8 g FASTIX®; for E2 5 mg: 300 mg E2+1.8 g FASTIX®; E2 20 µg: 1.3 mg E2+2.11 g FASTIX®; MPA 40 mg: 800 mg MPA +1.2 g FASTIX®; MFP 6 mg: 360 mg MFP +1.8 g FASTIX®. While the adhesive was still malleable, the mixture was pressed between two glass slides wrapped in greaseproof paper and separated by two 6 mm spacers. The steroid tablets were left to harden overnight at room temperature and cut in equal pieces with a 4 mm (Pg, E2 or MFP; pellet weight: 0.03 g) or 6 mm (MPA; pellet weight: 0.1 g) diameter hollow punch (Balbico, Buenos Aires, Argentina). Control pellets, with no hormone, were prepared following the same procedure but with vehicle (FASTIX®) alone. Eventually, pellets with different amounts of steroids can be prepared by mixing X mg of steroid with FASTIX®, weighing the steroid tablet (Y) and cutting and weighing 1 pellet (Z) from the tablet. Then, the amount of steroid in each pellet is calculated using the following formula: Steroid in 1 pellet: Z*X/Y.

Forty milligram MPA pellets were either prepared as described above or purchased from Hormone Pellet Press (Shawnee Mission, KS, USA).

### Ovariectomy and Hormone Replacement

Ovariectomy was performed under ketamine/xylazine anesthesia (10 mg/1 mg per 100 g of body weight, respectively) and, following a one week wash-out period we implanted the HMSP subcutaneously (sc) using a trocar into the back of 2-month-old anesthetized mice. Mice were treated with HMSP (n = 4 per group) containing 20 µg E2, 5 mg E2, 5 mg Pg or control for 24 or 48 hours and, 7, 14 or 28 days. Control animals (n = 13) were implanted with vehicle-containing pellets. At each time point the mice were weighed and blood samples and vaginal smears for estrous cycle evaluation were collected. Animals were euthanized by CO_2_ inhalation; mammary glands and uteri were dissected, weighed, fixed in 10% buffered formalin, embedded in paraffin and stained with hematoxylin and eosin.

### Blood Sampling

Blood samples were collected under ketamine/xylazine anesthesia using the technique of submandibular bleeding followed by a terminal cardiac puncture procedure. Blood samples were allowed to clot overnight at 4°C, centrifuged at 1000 g for 5 minutes at 4°C, serum was collected and stored at −70°C until radioimmunoassay.

### Tumor Growth

C4-HD is a mammary carcinoma induced by MPA in a BALB/c female mouse. C4-HI is a hormone independent (HI) variant derived from C4-HD, capable of growing in untreated mice and regressing with antiprogestin treatment [13]. Both variants express estrogen receptor alpha (ERα) and progesterone receptors (PR) [13]. Tumors were maintained by serial sc transplantations into MPA-treated (C4-HD) or untreated (C4-HI) female mice. To evaluate tumor regression, when the tumors reached an approximate size of 50 mm^2^, the animals were treated with the antiprogestin MFP either by sc injections (solution dissolved in phosphate buffer: 12 mg/kg/day) [14] or in the form of HMSP (6 mg) implanted at the back of the animals. Tumor size was measured with a caliper.

### Radioimmunoassay (RIA)

All hormones were assayed by RIA. Serum E2 levels were measured using a Double Antibody RIA kit (KE2D1– PIKE2D-9) provided by Siemens (Siemens USA, Los Angeles, CA) following the manufacturer’s instructions. Serum aliquots of 15 µl from the E2 20 µg group, and 15 µl of diluted serum (1/10) from E2 5 mg group were used in duplicate. The dilution was prepared with the zero calibrator provided by the kit. The protocol was adapted to 280 µl of final volume, considering the amount of serum that can be collected from one mouse, in accordance with the Guide for the Care and Use of Laboratory Animals [12]. Calibration curve: 1–200 pg/ml. Intra- and inter- assay coefficients of variation were 4.2 and 15.1%, respectively.

Pg was determined using an antibody provided by G. D. Niswender, and labeled hormone (progesterone [1,2,6,7 3H(N)]) was purchased from Dupont NEN (Boston, MA) [15]. Serum aliquots of 50 µl were used for extraction with hexane and then 500 µl of the resuspended extraction solution (2 ml) were used in duplicate for RIA assay. The lowest detectable concentration was 12.5 pg and intra- and inter-assay coefficients of variation were 7.5 and 11.9%, respectively.

Prolactin (Prl) was measured using mouse specific reagents provided by the National Institute of Diabetes and Digestive and Kidney Diseases (NIDDK), National Hormone and Pituitary Program (Dr. A. F. Parlow, Torrance, CA) [16]. Assays were performed using 10 µl of serum in duplicate. Results are expressed in terms of mouse Prl reference preparation RP3. Intra-assay and inter-assay coefficients of variation were 7.2 and 12.8%, respectively.

LH concentrations were determined using reagents provided by Dr. Parlow from the NIDDK [17]. Assays were performed using 75 µl of serum in duplicate. The lowest detectable concentration was 0.015 ng. Results were expressed in terms of mouse-LH standard (AFP 5306A). Intra- and inter-assay coefficients of variation were less than 8 and 11%, respectively.

### Uterine Epithelial Cell Height

In uterine sections endometrial epithelial cell height was measured from the apical (luminal) surface to the basement membrane. All measurements were made in areas where luminal folds were not present and care was taken to avoid measuring in sections that may have been cut obliquely. A representative section was selected for each animal, and the average of ten measurements per section was used in the calculations. Photographs were taken with a Nikon Eclipse E800 Microscope and measurements were made using Image Quant software at 400X magnification.

### Immunohistochemistry

Tumor samples, mammary glands and uteri were fixed in 10% buffered formalin and embedded in paraffin. Sections were dewaxed in xylene, rehydrated through graded ethanol, and treated for 30 minutes with 10% H_2_O_2_ in 70° ethanol to quench endogenous peroxidase activity. The slides were washed extensively with distilled water followed by PBS, three times for 5 minutes each time. The sections were then incubated in 5% albumin in PBS for 40 minutes and then reacted with PR (H-190, rabbit polyclonal IgG, Santa Cruz Biotech, CA) or ERα (MC-20, rabbit polyclonal IgG, Santa Cruz Biotech, CA) diluted 1∶100 in PBS for 24 h at 4°C. The slides were washed with PBS and incubated for 60 min at room temperature with anti-rabbit biotin-conjugated immunoglobulin (Vector Labs, San Francisco, CA), diluted 1∶250 in PBS, washed, and incubated for 30 minutes with the ABC complex, prepared according to the manufacturer’s directions (Vector Labs). The slides were thoroughly washed with PBS, and developed under microscopic control with liquid 3–3′diaminobenzidine and substrate chromogen system (Dako, Carpinteria, CA) following the manufacturer’s instructions.

### Statistical Analysis

Results are expressed as means ± S.E.M. Mean hormone serum concentration (Prl, E2 and LH) and uterine weight were analyzed by two-way ANOVA (effects of treatment and time) and differences among treatment groups were assessed by *Fisher* protected least-significant difference (LSD) and *Bonferroni post test*. One-way ANOVA was applied for serum Pg levels and means were compared by Fisher’s LSD test. *p*<0.05 was considered significant. Tumor growth curves were studied using regression analysis and slopes compared using one-way ANOVA followed by parallelism analysis.

## Results

### E2, Pg, LH and Prl Serum Levels after E2- or Pg-HMSP Implantation

**Figure 1:**
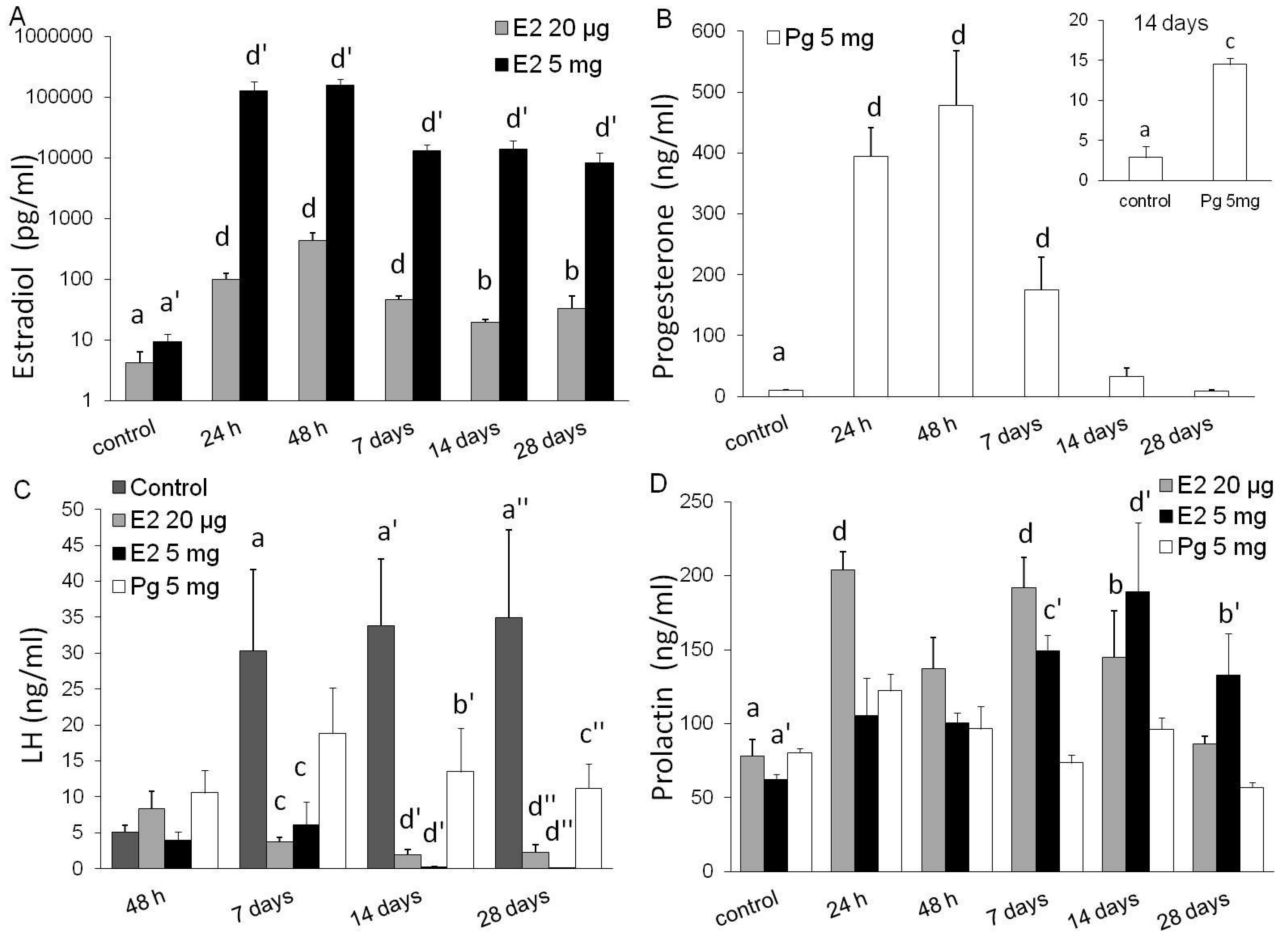
E2, Pg, LH and Prl serum levels after E2- or Pg-HMSP implantation. Effect of E2- or Pg-HMSP on serum levels of E2 (**A**), Pg (**B**), LH (**C**) or Prl (**D**) in ovariectomized adult mice treated for up to 28 days with the HMSP (n = 4–6/group). Values represent means ± S.E.M. Values with different letters are significantly different (a *vs.* b or a′ *vs.* b′: p<0.05; a *vs.* c or a′ *vs.* c′: p<0.01; and a *vs.* d or a′ *vs.* d′ or a′′ *vs.* d′′: p<0.001). Two-way ANOVA (treatment by time) with *post-hoc* Fisher’s tests was performed for LH (p interaction = 0.0089), Prl (p interaction = 0.011) and E2 (p interaction = 0.00018); and one-way ANOVA with *post-hoc* Fisher’s tests in Pg (p time <0.0001). Inset: Student t test (p<0.01).

Significant levels of E2 and Pg were detected already 24 h after pellet implantation (Figure 1A and B). High E2 levels were maintained throughout the experiment with the 5 mg E2 pellets, while circulating E2 values decreased from day 7 to day 14 with the low-dose pellets, although the values were still significantly higher than control levels even at day 28. Although both E2 curves followed the same shape, two-way ANOVA indicated that there was a dose-dependent effect and E2 values were lower with the low E2 dose compared with the high E2 dose from 24 h onwards [Figure 1A; p interaction (treatment × time) = 0.00018, and p<0.0004 for E2 levels at every time point from 24 h onwards].

Circulating values of Pg showed a rapid increase at 24 h, peaking at 48 h post pellet implant. Thereafter serum Pg levels decreased gradually, but were still higher than controls up to 14 days (Figure 1B & inset). In the comprehensive analysis, differences in Pg levels at day 14 were not significant probably due to the increased variance caused by the high values measured during the early time points, although values at least doubled those of the control. Therefore, new trials were conducted focusing on the detection of Pg on day 14, obtaining significantly higher levels than in the control group (Figure 1B inset; p<0.01).

In the case of LH analysis, control serum levels were not pooled, as in the previous analysis, because LH values changed with time post ovariectomy and thus, low levels were found during the first time point. Both E2 doses effectively downregulated the increase in serum LH levels evoked by ovariectomy from days 7 to 28 (Figure 1C). On the other hand, highest Pg levels (Figure 1B) were obtained when LH had not yet increased in response to ovariectomy (24 and 48 h) but thereafter, Pg-HMSP-pellets inhibited LH levels (even though not significantly at 7 days). The degree of inhibition was lower than for E2, as expected (Figure 1C).

It is well established that E2 enhances Prl secretion [7] and E2-HMSP-pellets (20 µg and 5 mg) increased Prl levels, thus proving the efficiency of these pellets. The low dose pellet induced higher Prl levels at 24 h, 7 and 14 days, while the high-dose E2 pellet induced Prl secretion from day 7 onwards, exerting its maximum effect on day 14 (Figure 1D). On the other hand, Pg-HMSP did not increase Prl levels at any time point, as expected.

### Uterine Weight and Vaginal Smears after E2- or Pg-HMSP Implantation

**Figure 2:**
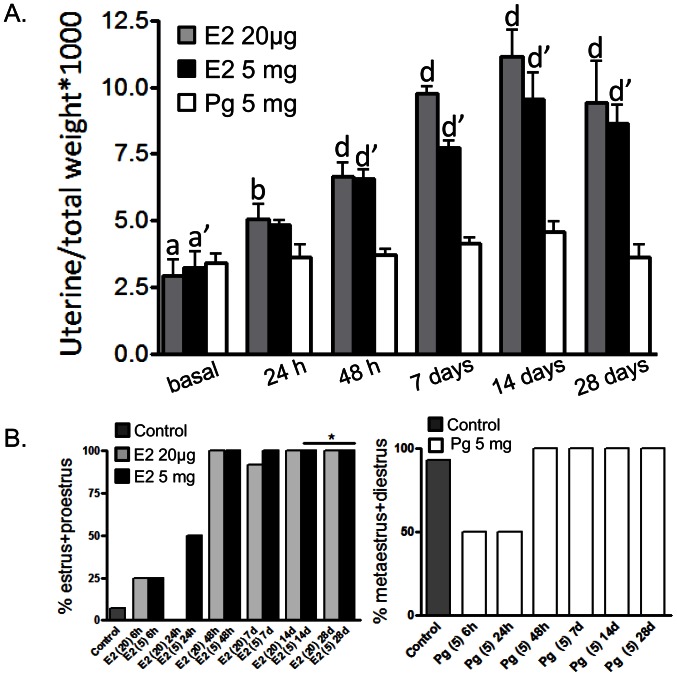
Uterine weight and vaginal smears after E2- or Pg-HMSP implantation. (**A**) Ratio between uterine and total animal weight of mice treated for 0, 24 and 48 h and 7, 14 and 28 days with E2- (20 µg), E2- (5 mg) and Pg- (5 mg) HMSP. Values with different letters are significantly different (a *vs.* b: p<0.05; a *vs.* d or a′ *vs.* d′: p<0.001). (**B**) Percent of animals in proestrus and estrus **(left)** or metaestrus and diestrus **(right)** following treatment with E2-HMSP **(left)** or Pg-HMSP **(right)** for different time points. *: with sustained administration of E2 pure estrus were not observed: the smears revealed a mixture of cornified epithelial cells with leukocyte invasion probably due to the accentuation of normal physiological changes induced by extreme hormonal stimulation [32].

Uterine weight and vaginal smears were used to monitor the pathophysiologic effects of the E2- and Pg-HMSP. Both E2 pellets induced a significant increase in uterine weight as compared to control- or Pg-treated animals (Figure 2A). Low doses of E2 were sufficient to exert a maximal effect. Vaginal cornification was observed in E2-treated mice within 48 h while Pg induced a metestrous-diestrous state (Figure 2B). No evidences of pellet rejection (edema or swelling) or signs of toxicity (body weight and histopathological damage) were found at necrospy when mice were treated with control handmade pellets (not shown).

### Effect of E2- or Pg-HMSP in Uterine and Mammary Gland Histology

**Figure 3:**
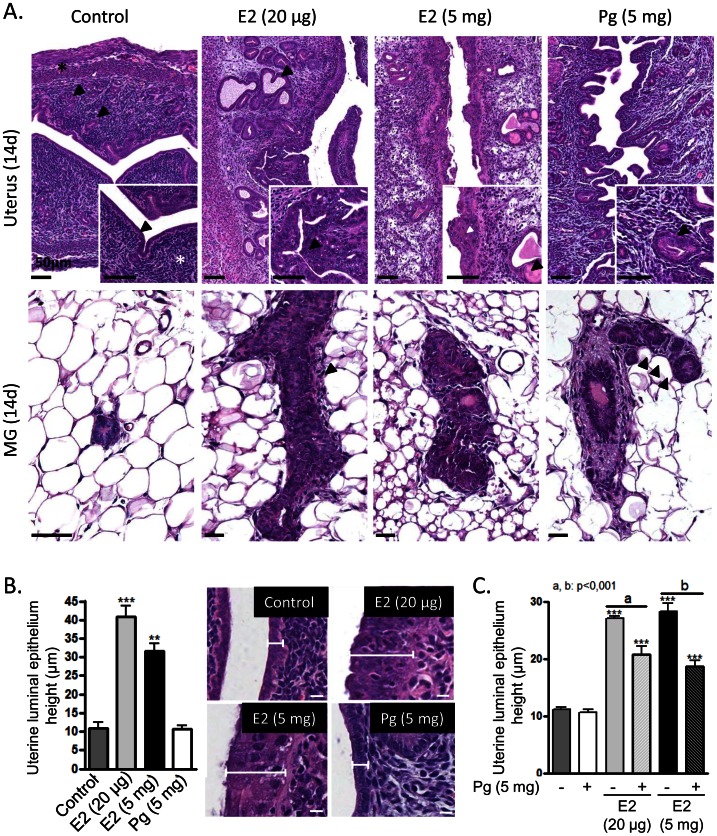
Effect of E2- or Pg-HMSP in uterine and mammary gland morphology. (**A**) Histological features of 5- µm-thick sections of uteri and mammary glands from mice treated for 14 days with control, E2- (20 µg), E2- (5 mg) and Pg- (5 mg) HMSP. Mice were ovariectomized followed by a 7 day washout period and treated for 14 days. **Uterus**. **Control:** The endometrium is lined by a low cylindrical epithelium (arrow head, inset); the endometrial stroma is densely packed (star, inset), with embedded small endometrial glands (arrow heads). The star in the main picture shows the thinned myometrial wall. **E2 20 µg:** Well-developed endometrial glands with proteinaceous material inside (arrow heads). The surface epithelium is locally stratified, composed of more than one layer of cells (arrow heads, inset). **E2 5 mg:** The surface epithelium is irregular and composed of one or more cell layers (inset, white arrow head) with focal apoptosis, the lumen discloses plugs of coagulated secretion (inset, arrow head) and mononuclear cells. **Pg 5 mg:** The cells lining the glandular structures are cylindrical and the nuclei are basally located (inset, arrow head). **Mammary gland**. **E2 20 µg:** Well-developed ductal structure lined by a stratified cuboidal epithelium; a thin stromal layer is evident in this picture (arrow head). **Pg 5 mg:** Ductal structure showing signs of initial lobular development (arrow heads). Magnification bar: 50 µm. (**B**) Effect of E2- and Pg-HMSP on luminal epithelium height following 14 days of pellet implantation to ovariectomized mice. Bar: 10 µm. ***: p<0.001; **: p<0.01. (**C**) Effect of co-administration of E2- and Pg-HMSP on luminal epithelium height following 7 days of pellet implantation to ovariectomized mice. ***: p<0.001; a *vs*. b: p<0.001.

The effects of E2 and Pg treatments in the uteri and mammary glands were evident in the H&E-stained sections (Figure 3A). By day 14, the endometrium of the untreated ovariectomized mice was lined by a low cylindrical epithelium with scanty cytoplasm and luminal-located nuclei; the endometrial stroma was dense, with embedded small endometrial glands with no signs of secretion (Figure 3A). Treatment with both doses of E2-HMSP resulted in a tortuous endometrium lined by a tall columnar epithelium, pseudostratification and an increase in the size and number of glands. Endometrial glands were filled with a proteinaceous material. In the uterine stroma, decidualization and edema was appreciated. All these changes were typically dose-dependent (Figure 3A). The endometrium from Pg treated mice was composed of well-developed and convoluted glands with local pseudopapillar images, deeply immersed in a well-vascularized and edematous stroma (Figure 3A). The untreated mammary glands displayed poorly developed glandular structures, lined by cuboidal epithelium lying directly upon the adipose tissue, with seemingly no ductal wall development. As expected, E2-treatment induced a well-developed ductal structure lined with a stratified cuboidal epithelium while Pg triggered lobular development (Figure 3A).

### Effect of E2- or Pg-HMSP in Uterine Luminal Epithelium Height

Measurements of uterine luminal epithelial height following treatments with low and high dose E2- and/or Pg-HMSP are shown in Figures 3B and C. As expected, both E2 doses significantly increased luminal epithelial height following 7 and 14 days of treatment. In order to test if Pg-HMSP could reduce E2 induced uterotrophy we administered Pg combined with high dose and low dose E2 pellets during 7 days. The co-administration of 5 mg Pg-HMSP significantly reduced (although above control levels) the effects of high and low E2 pellets (Figure 3C). Pg alone did not affect uterine epithelial height (Figures 3B and C).

### Effect of E2- or Pg-HMSP in ERα and PR Expression in the Uteri and Mammary Glands

**Figure 4:**
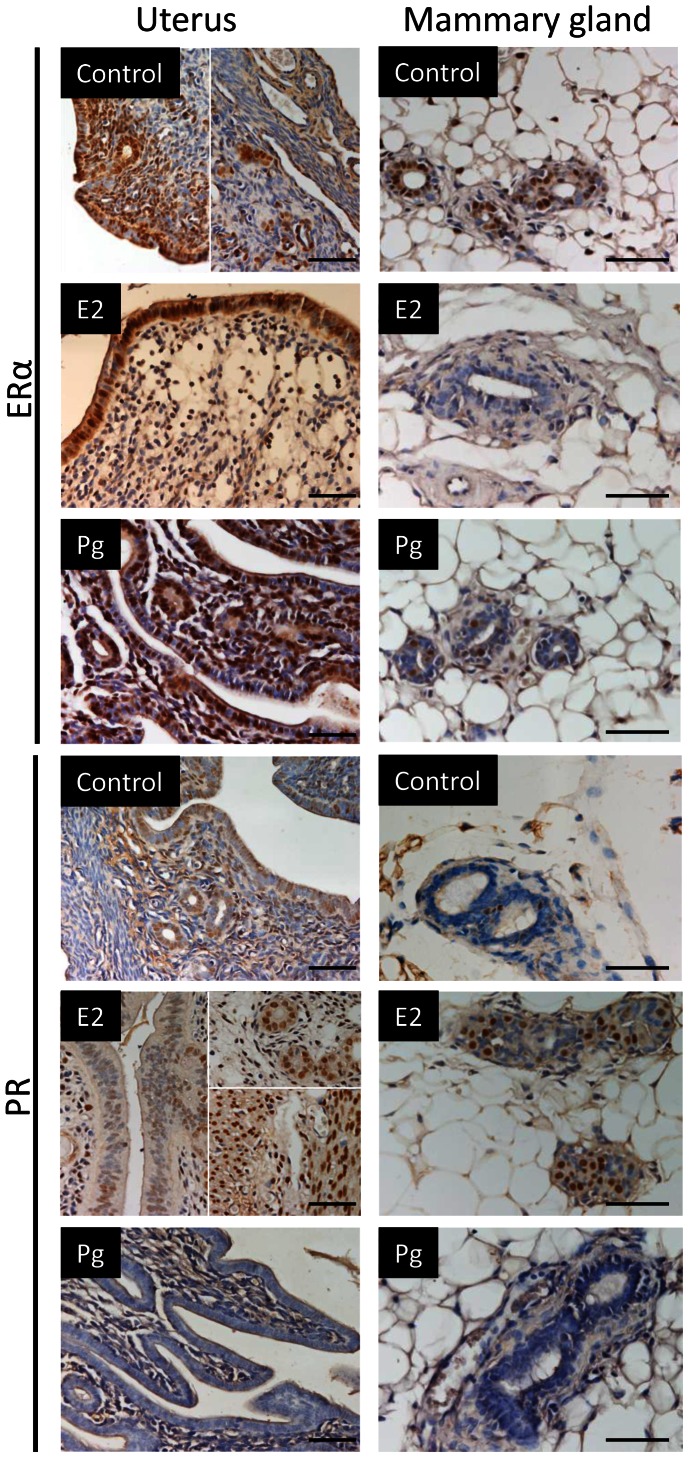
ERα and PR expression in reproductive tissues. Immunohistochemical expression of ERα **(upper panel)** and PR **(lower panel)** in uteri **(left)** and mammary glands **(right)** treated for 7 days with E2 (5 mg), Pg (5 mg) or vehicle alone. Bar: 50 µm.

We examined E2 and Pg regulation of ERα and PR expression in uteri and mammary glands by immunohistochemistry (Figure 4).

Control uteri from mice administered vehicle alone revealed strong nuclear staining within the epithelial and stromal compartment, with almost no staining in the myometrium. Treatment with E2 resulted in a strong ERα staining within the luminal epithelial compartment and a weak staining in the stroma. ERα staining following 7 days of Pg treatment revealed similar results as those obtained with control mice (Figure 4).

Immunohistochemical analysis of PR staining in uteri from control ovariectomized mice revealed an intense staining in the epithelial compartment while the myometrium showed almost no staining. E2 treatment with 5 mg HMSP resulted in a strong staining in the glandular epithelium and in the myometrium. Pg treatment induced a marked downregulation in PR expression in the uteri (Figure 4).

In the mammary gland, after 7 days of E2 treatment with 5 mg HMSP, ERα expression was downregulated in the mammary ducts while no significant changes were observed following Pg treatment. Conversely, E2 induced while Pg downregulated PR expression within the mammary ducts (Figure 4).

### Effect of MPA- or MFP-HMSP on Mammary Tumor Growth

**Figure 5:**
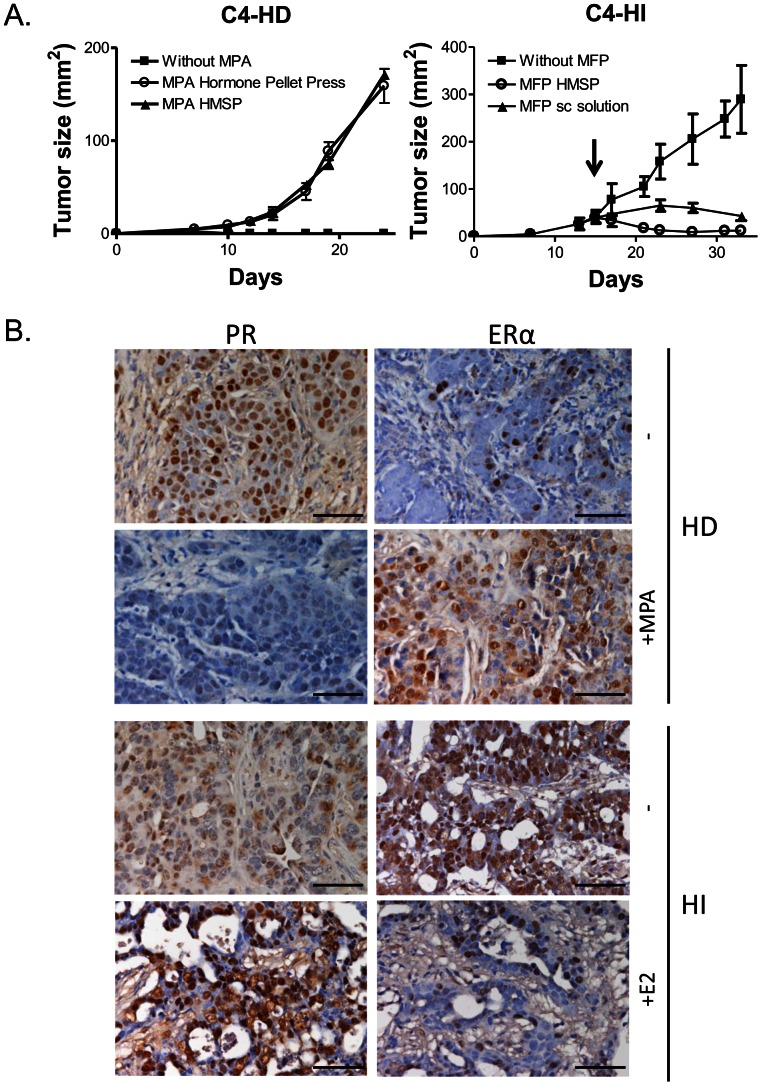
Tumor growth and hormone receptor regulation in endocrine treated murine mammary carcinomas tumors. (**A**) Effects of MPA or MFP on tumor growth. **(Left)** BALB/c mice were sc transplanted in the inguinal flank with C4-HD mammary carcinomas and simultaneously MPA- (40 mg) HMSP or Hormone Pellet Press pellets were sc implanted into the back of the mice (N = 4–6/group). Treatments began when tumors were transplanted. **(Right)** Mice bearing C4-HI mammary carcinomas were treated with 6 mg MFP-HMSP (N = 3), MFP solution (12 mg/kg/day; N = 3) or left untreated (N = 6). Treatments began when tumors reached approximately 50 mm^2^ (arrow). Tumor sizes are expressed as mean ± SD. (**B**) Immunohistochemical expression of ERα (**right**) and PR (**left**) in C4-HD (**upper panel**) and C4-HI (**lower panel**) mammary carcinomas. C4-HD carcinomas were treated with MPA and when tumors reached approximately 50 mm^2^, the pellet was removed for 48 h in untreated tumors. C4-HI-bearing mice were treated for 72 h with E2 (5 mg) once the tumors reached 50 mm^2^. Bar: 50 µm.

Finally, we compared the effectiveness of HMSP with the biological effect of other synthetic compounds such as the progestin MPA and the antiprogestin MFP in an *in vivo* system (Figure 5A). We found that the hormone dependent tumor (C4-HD), which is unable to grow in the absence of the progestin [13], grew at a similar rate with the MPA-HMSP pellets or with the commercially available MPA pellets. Moreover, the growth of the hormone independent tumor variant (C4-HI) which regresses after antiprogestin treatment [18], was inhibited by MFP administered either as HMSP (6 mg) or as sc suspension (12 mg/kg/day).

### Effect of MPA- or E2-HMSP on Hormone Receptor Regulation in Mammary Tumors

Next, we assayed the effect of MPA- and E2-HMSP on ERα and PR expression in mammary tumors. E2 induced HI tumor regression and, in the absence of progestin administration HD carcinomas regressed (Figure 5B) [19], [20]. We found that MPA treatment downregulated PR and upregulated ERα expression in the epithelial compartment of C4-HD mammary tumors in agreement with previous results with MPA depot [Figure 5B, [20]]. Moreover, E2 treatment induced a strong PR staining in carcinoma cells while downregulating ERα expression in C4-HI mammary tumors (Figure 5B).

## Discussion

In this study we demonstrate that the HMSP elaborated with FASTIX® adhesive provides a useful method for easy delivery of steroids to mice. These pellets combine the advantages of administering steroids over rather long periods of time, at a low cost and without daily attention. FASTIX® adhesive contains PDMS, the same component present in Silastic® glue, and is easily accessible and affordable for scientists. Moreover, no adverse effects of FASTIX® were observed in any of the endpoints measured in this study. This product is also sold in other countries such as Spain and several countries in Latin America under the same name.

We found significant levels of E2 and Pg within 24 h of initial placement of the implant which lasted for over 28 or 14 days, respectively. Elevated steroids resulted in an inhibition of LH release (in E2- and Pg-treated mice), an enhanced secretion of Prl and an increase in uterine weight (in E2-treated mice) in accordance with the results reported by us [21] and other authors using different techniques [7], [22], [23]. The concentration of E2 released by HMSP can be easily manipulated to obtain a controlled delivery of the steroid. Significant E2 release was measured throughout the experiment while Pg levels declined substantially as already reported in other models [24], [25], probably due to the short half-life of natural Pg [26].

The low- and high- dose E2 pellets induced a significant increase in Prl levels within 24 h and 7 days, respectively, reaching their maximum values at day 7 and 14, indicating a shift in the serum Prl secretion curve depending on the E2 dose. Similar high Prl levels were achieved by both doses. Similar to what we present here, Bronson et al [7] reported no significant differences between high and low E2 doses in ovariectomized mice treated for 8 days. Furthermore, we and others have shown that very high estrogen levels, unlike lower dosages, can inhibit or have no effect on Prl release in mice [27], [22]. One possibility is that high estradiol doses may interfere with the actions of hypothalamic-releasing factors leading to storage of prolactin within the lactotrophs.

In this study we demonstrated that E2- and Pg-HMSP exerted the expected biological effects on: vaginal smears (by inducing a continuous estrus state), uterine weight, uterine luminal epithelial height and ERα and PR regulation in the uteri and mammary gland, as already reported in similar models with commercial pellets [28], [29]. Moreover, combined administration of E2 and Pg in ovariectomized mice reduced uterine luminal epithelial as compared to E2 alone, being the ultimate functional proof of the effectiveness of this delivery system.

We have previously shown that the C4-HD mammary carcinoma grows only in progestin-treated mice while the C4-HI tumor grows in treated or untreated mice. Both variants express ERα and PR, and antiprogestins such as MFP induce tumor regression [13]. In this report, we validated the effectiveness of our HMSP by comparing the effect on HD and HI tumor growth with commercially available sc MPA pellets or daily injections of MFP solution, that have proven to be effective in inducing or inhibiting tumor growth, respectively [13]. Pg HMSP induced lobular differentiation in mammary glands from ovariectomized mice [30], [31]. We found that HMSP induced similar biological effects on tumor growth and mammary gland histology as the other validated methods used to administer steroids, demonstrating that this is an effective method for delivering steroids to mice.

In this first pharmacological phase, we have focused in evaluating steroid release and, as a result, the well-established effects on hormone regulation, uterine weight and histological features in reproductive tissues. In a second phase, we will perform experiments to try to achieve physiological endocrine profiles with HMSP.

### Conclusions

We provide clear evidence that PDMS-based adhesives such as FASTIX® can be used to prepare highly effective and low cost pellets. This methodology is similar to Silastic glue pellets with the advantage that PDMS adhesives have a lower cost and can be easily obtained in any store for that category. It is useful not only for natural but also for synthetic steroids such as medroxyprogesterone acetate and MFP, providing a new way to deliver steroids to mice.
